# Endogenous Viral Sequences from the Cape Golden Mole (*Chrysochloris asiatica*) Reveal the Presence of Foamy Viruses in All Major Placental Mammal Clades

**DOI:** 10.1371/journal.pone.0097931

**Published:** 2014-05-16

**Authors:** Guan-Zhu Han, Michael Worobey

**Affiliations:** Department of Ecology and Evolutionary Biology, University of Arizona, Tucson, Arizona, United States of America; University of Athens, Medical School, Greece

## Abstract

Endogenous retroviruses provide important insights into the deep history of this viral lineage. Endogenous foamy viruses are thought to be very rare and only a few cases have been identified to date. Here we report a novel endogenous foamy virus (CaEFV) within the genome of the Cape golden mole (*Chrysochloris asiatica*). The identification of CaEFV reveals the presence of foamy virus in the placental mammal superorder Afrotheria. Phylogenetic analyses place CaEFV basal to other foamy viruses of Eutherian origin, suggesting an ancient codivergence between foamy virus and placental mammals. These findings have implications for understanding the long-term evolution, diversity, and biology of retroviruses.

## Introduction

Foamy viruses are complex retroviruses, which are typically nonpathogenic and infect a variety of placental mammals, including primates, cats, cows, bats, and horses [1], [2]. Retroviruses can integrate into host genomes as endogenous retroviruses (ERVs), which provide ‘molecular fossils’ for studying their deep history and their relationships with their hosts [3]. While ERVs are common in vertebrate genomes [4], endogenous foamy virus-like elements are thought to be very rare [5]–[7]. To date, endogenous foamy viruses have been found only within the genomes of the sloths [5], aye-aye [6], coelacanth [7], zebrafish [8], platyfish [9], and cod [9]. The discovery of endogenous foamy virus-like elements in coelacanth suggested that foamy viruses and their vertebrate hosts have likely codiverged for more than 407 million years [7].

The steady accumulation of additional animal genome sequences currently offers a great opportunity to discover novel endogenous foamy virus-like elements, which could provide important insights into the evolutionary history and biology of foamy viruses. Here we report the discovery of an endogenous foamy virus within the genome of a small, insectivorous mammal native to southwestern South Africa, the Cape golden mole (*Chrysochloris asiatica*), which we designate ‘*Chrysochloris asiatica* endogenous foamy virus’ (CaEFV). This finding provides strong evidence that foamy viruses were already present in the most recent common ancestor of all placental mammals ∼100 million years ago.

## Methods and Materials

### Genome screening

All whole-genome shotgun sequences from animals available from NCBI were screened for endogenous foamy viruses using the TBLASTN algorithm and the protein sequences of representative foamy viruses. The following representative foamy viruses were used: bovine foamy virus (NC_001831), equine foamy virus (NC_002201), feline foamy virus (NC_001871), *Rhinolophus affinis* foamy virus (JQ814855), spider monkey simian foamy virus (EU010385), gorilla simian foamy virus (HM245790), chimpanzee simian foamy virus (NC_001364), macaque simian foamy virus (NC_010819), and African green monkey simian foamy virus (NC_010820).

### Phylogenetic analysis

Protein sequences were aligned using MUSCLE [10] and then manually edited (Dataset S1 and Dataset S2). We used Gblocks 0.91b to exclude poorly aligned regions from the analyses [11]. To determine the relationship between CaEFV and other retroviruses, a phylogenetic tree was reconstructed with the conserved Pol protein regions using a neighbor-joining method implemented in MEGA5.2 [12]. Node supports were evaluated via nonparametric bootstrap analyses with 1000 replicates. To evaluate the relationship between CaEFV and other endogenous and exogenous foamy viruses, a phylogenetic tree was reconstructed with the conserved Env protein regions using a Bayesian approach. The Bayesian analysis was performed with MrBayes 3 [13] using 1,000,000 generations in four chains, sampling posterior trees every 100 generations. The first 25% of the posterior trees were discarded.

## Results and Discussion

**Figure 1:**
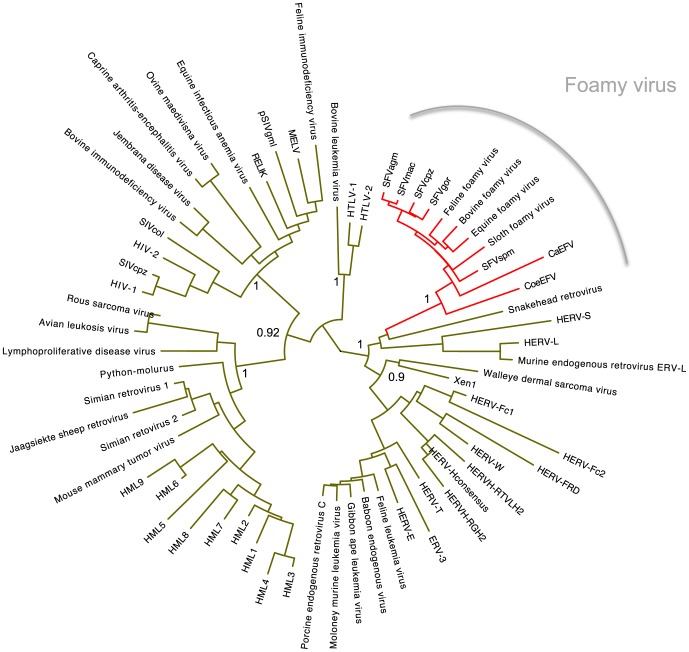
Phylogenetic analysis of CaEFV and other retroviruses. The phylogenetic tree was reconstructed based on conserved regions of CaEFV and other representative retrovirus Pol proteins using the neighbor-joining method with 1,000 bootstrap replicates. The node labels are bootstrap values. Only selected bootstrap values are shown. The foamy virus clade is highlighted in red.

As expected, TBLASTN screening of all animal whole-genome shotgun sequences available from NCBI detected several previously identified endogenous foamy virus-like elements in the genomes of the sloth, the aye-aye, and the coelacanth [5]–[7]. However, we also identified highly significant matches to foamy virus proteins (Pol and Env proteins) within a contig (contig151999) of the Cape golden mole genome. The contig151999 contains a partial foamy virus (*pol* and *env* genes) insertion (Table 1). Phylogenetic analysis of this sequence, which we refer to as ‘CaEFV’ (*Chrysochloris asiatica* endogenous foamy virus), along with various other retroviruses shows that CaEFV groups with foamy viruses with robust support (Fig. 1). Moreover, both BLASTP and PSI-BLAST that is capable of detecting distant relationship between proteins [14] using CaEFV as a query only found significant hits from foamy viruses, but not from other retroviruses (E value threshold of 0.01; Table S1 and Table S2). These results confirm that CaEFV is indeed an endogenous foamy virus.

**Table 1:** The Cape golden mole genome contig the match foamy virus gene sequences.

Contig	Position	Genomic region represented	Best hit (accession No.)	Identity	BLASTP E-value
contig151999	609–3534	*pol*	Spider monkey foamy virus (ABV59399)	42%	0.0
	3476–6371	*env*	Bovine foamy virus (AFR79245)	29%	2E-114

Because we only identified a single copy of CaEFV and did not find long terminal repeats (LTRs), we cannot estimate the insertion time of CaEFV into the Cape golden mole genome. However, the presence of multiple premature stop codons suggests the invasion occurred long time ago (see ref 6 for discussion of a similar case) (Dataset S3).

**Figure 2:**
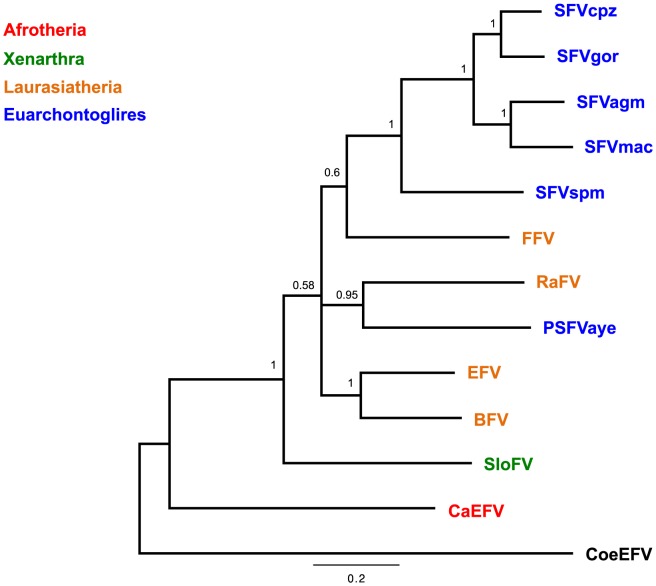
Phylogenetic analysis of CaEFV and other endogenous and exogenous foamy viruses. The phylogenetic tree is 50% majority-rule consensus tree reconstructed based on conserved regions of foamy virus Env proteins using MrBayes 3. The node labels are posterior probabilities. Branch lengths are in expected changes per site. The viruses are colored according to the superorder their hosts belong to. BFV, bovine foamy virus; EFV, equine foamy virus; FFV, feline foamy virus; RaFV, *Rhinolophus affinis* foamy virus; SFVspm, spider monkey simian foamy virus; SFVgor, gorilla simian foamy virus; SFVcpz, chimpanzee simian foamy virus; SFVmac, macaque simian foamy virus; SFVagm, African green monkey simian foamy virus; SloEFV, sloth endogenous foamy virus; PSFV, aye-aye prosimian foamy virus; CoeEFV, coelacanth endogenous foamy-like virus. This consensus tree is depicted with the CoeEFV sequence as the outgroup, but it is an unrooted phylogeny and there is thus no posterior probablity associated with the node connecting the CaEFV sequence with the other mammalian ones.

To further determine the relationship between CaEFV and other endogenous and exogenous foamy viruses, we reconstructed phylogenetic trees using conserved regions of the Env protein. Our phylogenetic analysis shows that CaEFV is basal to other exogenous and endogenous foamy viruses of Eutherian origin (Fig. 2). Placental mammals can be divided into four major clades: Afrotheria (e.g. golden moles, tenrecs, elephants, aardvarks), Xenarthra (e.g. anteaters, tree sloths, armadillos), Laurasiatheria (e.g. bats, whales, hoofed mammals, carnivores), and Euarchontoglires (e.g. rodents, lagomorphs, primates). The latter two are phylogenetically monophyletic and are jointly deemed Boreoeutheria [15]. The Cape golden mole belongs to the superorder Afrotheria, while the sloths belong to the superorder Xenarthra. Previous studies reveal that simian foamy viruses have codiverged with Old World primates for more than 30 million years [16]. Furthermore, analyses of coelacanth endogenous foamy virus suggest foamy viruses and their vertebrate hosts are likely to have codiverged for more than 407 million years [7]. Although the relationship of Afrotheria, Xenarthra, and Boreoeutheria remains poorly resolved [17], [18], the basal position of CaEFV is compatible with the ancestral codivergence of foamy viruses and their placental mammal hosts, given that the discovery of an Afrotherian foamy virus indicates that all major placental mammal lineages were infected. This, in turn, suggests continuous presence of mammalian foamy viruses since the time of the most recent common ancestor of all placental mammals, estimated at ∼100 million years ago [19].

The foamy virus phylogeny does not exactly match the species phylogeny ([14] and references therein). However, some of the key nodes on the viral phylogeny have very low posterior probabilities (Fig. 2). Nevertheless, the monophyletic grouping of bat foamy virus and aye-aye endogenous foamy virus with strong support suggests a putative host-jumping event between major placental mammal clades. But the exact scenario remains obscure, due to the low sampling coverage and the uncertainty of the key nodes in the phylogenetic trees.

Exogenous foamy viruses have been found exclusively in the superoder Laurasiatheria (such as bats, horses, cats, cows) and Euarchontoglires (such as primates) [1], [2]. The identification of CaEFV establishes the historical presence of foamy virus in the superorder of Afrosoricida. It would be of considerable interest to test for the presence of exogenous foamy viruses in this and other mammalian species outside of the two superorders known to harbor extant exogenous foamy viruses. Our analyses of endogenous foamy viruses extend their known host range to the superorder Afrotheria of placental mammals, in addition to previous evidence in Xenarthra as well as several fish species [5]–[9]. Therefore, foamy virus appears to be more widely distributed than previously thought [1], [2], [5]–[9]. More work is needed to characterize the diversity and distribution of foamy viruses; however, this additional evidence lends support to the idea that this retroviral lineage can be traced back more than 100 million years in mammals alone.

## Supporting Information

Table S1 **BLASTP results using CaEFV Env protein as a query (E value threshold of 0.01).**
(DOCX)

Table S2 **PSI-BLAST results using CaEFV Env protein as a query (E value threshold of 0.01).**
(DOCX)

Dataset S1 **The conserved region of Pol proteins of CaEFV and other retroviruses.**
(FAS)

Dataset S2 **The conserved region of representative endogenous and exogenous foamy virus Env protein.**
(FAS)

Dataset S3 **Amino acid sequences of CaEFV Pol and Env proteins.**
(FAS)
