# Supplementary material for: Endogenous Viral Sequences from the Cape Golden Mole (Chrysochloris asiatica) Reveal the Presence of Foamy Viruses in All Major Placental Mammal Clades
Source: PLoS One. 2014 May 16;9(5):e97931. doi: 10.1371/journal.pone.0097931 (PMC4024004; doi:10.1371/journal.pone.0097931)
Supplement: Table S1 — BLASTP results using CaEFV Env protein as a query (E value threshold of 0.01). (DOCX) [file pone.0097931.s001.docx]

**Table S1 BLASTP results using CaEFV Env protein as a query (E value threshold of 0.01)**

| **Description [taxon]** | **Max score** | **Total score** | **Query cover** | **E value** | **Identity** | **Accession** |
| --- | --- | --- | --- | --- | --- | --- |
| envelope protein [Bovine foamy virus] | 386 | 386 | 96% | 2.00E-114 | 29% | AFR79245.1 |
| envelope protein [Bovine foamy virus] | 386 | 386 | 96% | 3.00E-114 | 29% | AFR79240.1 |
| envelope protein [Bovine foamy virus] | 378 | 378 | 96% | 3.00E-111 | 30% | AAN08117.1 |
| envelope protein [Bovine foamy virus] envelope protein [Bovine foamy virus] | 377 | 377 | 96% | 4.00E-111 | 30% | NP_044930.1 |
| env polyprotein - human foamy virus >emb\|CAA29086.1\| unnamed protein product [Human spumaretrovirus] >gb\|AAA46123.1\| envelope protein [Human spumaretrovirus] | 372 | 372 | 99% | 3.00E-109 | 29% | VCLJSP |
| RecName: Full=Envelope glycoprotein gp130; AltName: Full=Env polyprotein; Contains: RecName: Full=Leader peptide; Short=LP; AltName: Full=Env leader protein; Short=Elp; AltName: Full=gp18LP; Contains: RecName: Full=Surface protein; Short=SU; AltName: Full=Glycoprotein 80; Short=gp80; Contains: RecName: Full=Transmembrane protein; Short=TM; AltName: Full=Glycoprotein 48; Short=gp48 >gb\|AAB48113.1\| envelope protein [Human spumaretrovirus] | 372 | 372 | 99% | 4.00E-109 | 29% | P14351.2 |
| envelope protein [Feline foamy virus] | 370 | 370 | 99% | 3.00E-108 | 29% | BAB33175.1 |
| env [Human foamy virus] >emb\|CAA68994.1\| env [Human foamy virus] >emb\|CAA69000.1\| env [Human foamy virus] | 369 | 369 | 99% | 7.00E-108 | 29% | CAA69004.1 |
| Env [equine foamy virus] >gb\|AAF64415.1\|AF201902_3 Env [equine foamy virus] | 365 | 365 | 96% | 2.00E-106 | 29% | NP_054717.1 |
| envelope protein [Simian foamy virus] >sp\|Q87041.1\|ENV_SFVCP RecName: Full=Envelope glycoprotein gp130; AltName: Full=Env polyprotein; Contains: RecName: Full=Leader peptide; Short=LP; AltName: Full=Env leader protein; Short=Elp; AltName: Full=gp18LP; Contains: RecName: Full=Surface protein; Short=SU; AltName: Full=Glycoprotein 80; Short=gp80; Contains: RecName: Full=Transmembrane protein; Short=TM; AltName: Full=Glycoprotein 48; Short=gp48 >gb\|AAA19979.1\| env [Simian foamy virus] | 364 | 364 | 99% | 3.00E-106 | 28% | NP_056804.1 |
| env protein [Simian foamy virus] | 360 | 360 | 93% | 1.00E-104 | 31% | AFX98100.1 |
| Env [African green monkey simian foamy virus] >sp\|P27399.1\|ENV_SFV3L RecName: Full=Envelope glycoprotein gp130; AltName: Full=Env polyprotein; Contains: RecName: Full=Leader peptide; Short=LP; AltName: Full=Env leader protein; Short=Elp; AltName: Full=gp18LP; Contains: RecName: Full=Surface protein; Short=SU; AltName: Full=Glycoprotein 80; Short=gp80; Contains: RecName: Full=Transmembrane protein; Short=TM; AltName: Full=Glycoprotein 48; Short=gp48 >pir\|\|VCLJLK env polyprotein - simian foamy virus (type 3, strain LK3) | 359 | 359 | 93% | 2.00E-104 | 30% | YP_001956723.2 |
| envelope protein, partial [Simian foamy virus 3] | 359 | 359 | 93% | 2.00E-104 | 30% | AAA47798.1 |
| env [Squirrel monkey foamy virus] | 356 | 356 | 99% | 2.00E-103 | 29% | ADE05996.1 |
| envelope protein [Macaque simian foamy virus] | 355 | 355 | 93% | 6.00E-103 | 29% | AFA44810.1 |
| env [Spider monkey foamy virus] | 355 | 355 | 99% | 6.00E-103 | 28% | ABV59400.1 |
| env protein [Macaque simian foamy virus] | 352 | 352 | 93% | 7.00E-102 | 29% | AGM61339.1 |
| env protein [Simian foamy virus] | 352 | 352 | 99% | 7.00E-102 | 28% | AFX98085.1 |
| env protein [Simian foamy virus] | 351 | 351 | 99% | 2.00E-101 | 28% | AFX98080.1 |
| Env [Macaque simian foamy virus] >sp\|P23073.3\|ENV_SFV1 RecName: Full=Envelope glycoprotein gp130; AltName: Full=Env polyprotein; Contains: RecName: Full=Leader peptide; Short=LP; AltName: Full=Env leader protein; Short=Elp; AltName: Full=gp18LP; Contains: RecName: Full=Surface protein; Short=SU; AltName: Full=Glycoprotein 80; Short=gp80; Contains: RecName: Full=Transmembrane protein; Short=TM; AltName: Full=Glycoprotein 48; Short=gp48 | 351 | 351 | 93% | 2.00E-101 | 29% | YP_001961123.1 |
| env protein [Macaque simian foamy virus] | 350 | 350 | 93% | 6.00E-101 | 29% | AGM61337.1 |
| env protein [Simian foamy virus] | 349 | 349 | 94% | 1.00E-100 | 29% | AFX98095.1 |
| envelope protein [Simian foamy virus] | 347 | 347 | 93% | 6.00E-100 | 29% | AAA47794.1 |
| envelope glycoprotein [Simian foamy virus-orangutan] | 345 | 345 | 99% | 3.00E-99 | 27% | CAD67563.1 |
| env [Common marmoset foamy virus] | 334 | 334 | 99% | 2.00E-95 | 28% | ADE06001.1 |
| envelope protein [Simian foamy virus-gorilla] | 333 | 333 | 94% | 5.00E-95 | 29% | ADN65592.1 |
| env protein [Simian foamy virus] | 332 | 332 | 94% | 1.00E-94 | 29% | AFX98090.1 |
| env protein [Macaque simian foamy virus] | 331 | 331 | 93% | 3.00E-94 | 29% | AGM61343.1 |
| Env [Feline foamy virus] | 328 | 328 | 99% | 4.00E-93 | 28% | AGC11914.1 |
| Env [Feline foamy virus] | 327 | 327 | 99% | 6.00E-93 | 28% | AGC11909.1 |
| envelope protein [Feline foamy virus] | 317 | 317 | 99% | 3.00E-89 | 28% | BAB33174.1 |
| envelope protein [Feline foamy virus] | 311 | 311 | 97% | 2.00E-87 | 28% | CAD92801.1 |
| envelope protein [Feline foamy virus] | 311 | 311 | 97% | 3.00E-87 | 28% | BAB33173.1 |
| hypothetical protein FFV_gp1 [Feline foamy virus] >sp\|O56861.1\|ENV_FFV RecName: Full=Envelope glycoprotein gp130; AltName: Full=Env polyprotein; Contains: RecName: Full=Leader peptide; Short=LP; AltName: Full=Env leader protein; Short=Elp; AltName: Full=gp18LP; Contains: RecName: Full=Surface protein; Short=SU; AltName: Full=Glycoprotein 80; Short=gp80; Contains: RecName: Full=Transmembrane protein; Short=TM; AltName: Full=Glycoprotein 48; Short=gp48 >emb\|CAA70076.1\| env [Feline foamy virus] >emb\|CAA11582.1\| env [Feline foamy virus] | 311 | 311 | 97% | 3.00E-87 | 28% | NP_056915.1 |
| FeSFV envelope [Feline foamy virus] >gb\|AAC58532.1\| envelope protein [Feline foamy virus] >emb\|CAD92797.1\| envelope protein [Feline foamy virus] | 310 | 310 | 97% | 7.00E-87 | 28% | AAB38322.1 |
| envelope protein, partial [Bovine foamy virus] | 204 | 204 | 54% | 1.00E-52 | 29% | ABM55470.1 |
| envelope protein, partial [Bovine foamy virus] | 202 | 202 | 54% | 2.00E-52 | 29% | ABM55473.1 |
| envelope protein, partial [Bovine foamy virus] | 201 | 201 | 54% | 8.00E-52 | 29% | ABM55472.1 |
| envelope protein, partial [Bovine foamy virus] | 201 | 201 | 54% | 1.00E-51 | 29% | ABM55471.1 |
| envelope protein, partial [Bovine foamy virus] | 199 | 199 | 54% | 3.00E-51 | 29% | ABM55474.1 |
| envelope protein, partial [Human spumaretrovirus] | 181 | 181 | 32% | 2.00E-46 | 33% | AAF00493.1 |
| env protein, surface domain [Simian foamy virus] | 168 | 168 | 48% | 1.00E-40 | 29% | CAB54085.1 |
| envelope glycoprotein, partial [Rhinolophus affinis foamy virus 1] | 165 | 165 | 45% | 5.00E-40 | 27% | AFK85016.1 |
| env protein, surface domain [Simian foamy virus] | 166 | 166 | 48% | 5.00E-40 | 29% | CAB54083.1 |
| env protein, surface domain [Simian foamy virus] | 166 | 166 | 49% | 7.00E-40 | 28% | CAB54084.1 |
| env protein, surface domain [Simian foamy virus] | 165 | 165 | 48% | 1.00E-39 | 29% | CAB54086.1 |
| env protein, surface domain [Simian foamy virus] | 165 | 165 | 48% | 1.00E-39 | 28% | CAB54088.1 |
| env protein, surface domain [Simian foamy virus] | 165 | 165 | 48% | 2.00E-39 | 28% | CAB54118.1 |
| env protein, surface domain [Simian foamy virus] | 165 | 165 | 48% | 2.00E-39 | 28% | CAB54082.1 |
| env protein, surface domain [Simian foamy virus] | 164 | 164 | 48% | 3.00E-39 | 29% | CAB54093.1 |
| env protein, surface domain [Simian foamy virus] | 164 | 164 | 48% | 3.00E-39 | 28% | CAB54106.1 |
| env protein, surface domain [Simian foamy virus] | 163 | 163 | 49% | 5.00E-39 | 28% | CAB54087.1 |
| env protein, surface domain [Simian foamy virus] | 163 | 163 | 48% | 5.00E-39 | 28% | CAB54110.1 |
| env protein, surface domain [Simian foamy virus] | 163 | 163 | 48% | 5.00E-39 | 28% | CAB54101.1 |
| env protein, surface domain [Simian foamy virus] | 163 | 163 | 48% | 5.00E-39 | 28% | CAB54104.1 |
| env protein, surface domain [Simian foamy virus] | 163 | 163 | 48% | 6.00E-39 | 28% | CAB54124.1 |
| env protein, surface domain [Simian foamy virus] >emb\|CAB54090.1\| env protein, surface domain [Simian foamy virus] | 162 | 162 | 48% | 7.00E-39 | 29% | CAB54089.1 |
| env protein, surface domain [Simian foamy virus] | 162 | 162 | 48% | 8.00E-39 | 28% | CAB54121.1 |
| env protein, surface domain [Simian foamy virus] | 162 | 162 | 48% | 8.00E-39 | 28% | CAB54130.1 |
| env protein, surface domain [Simian foamy virus] | 162 | 162 | 48% | 9.00E-39 | 28% | CAB54119.1 |
| env protein, surface domain [Simian foamy virus] | 162 | 162 | 48% | 1.00E-38 | 28% | CAB54114.1 |
| env protein, surface domain [Simian foamy virus] | 162 | 162 | 48% | 1.00E-38 | 28% | CAB54128.1 |
| env protein, surface domain [Simian foamy virus] >emb\|CAB54117.1\| env protein, surface domain [Simian foamy virus] >emb\|CAB54120.1\| env protein, surface domain [Simian foamy virus] >emb\|CAB54125.1\| env protein, surface domain [Simian foamy virus] >emb\|CAB54129.1\| env protein, surface domain [Simian foamy virus] | 162 | 162 | 48% | 1.00E-38 | 28% | CAB54099.1 |
| env protein, surface domain [Simian foamy virus] | 162 | 162 | 48% | 1.00E-38 | 28% | CAB54126.1 |
| env protein, surface domain [Simian foamy virus] | 162 | 162 | 48% | 1.00E-38 | 28% | CAB54102.1 |
| env protein, surface domain [Simian foamy virus] | 162 | 162 | 48% | 1.00E-38 | 28% | CAB54122.1 |
| env protein, surface domain [Simian foamy virus] | 162 | 162 | 48% | 1.00E-38 | 28% | CAB54127.1 |
| env protein, surface domain [Simian foamy virus] | 162 | 162 | 48% | 1.00E-38 | 28% | CAB54096.1 |
| env protein, surface domain [Simian foamy virus] | 162 | 162 | 48% | 1.00E-38 | 28% | CAB54115.1 |
| env protein, surface domain [Simian foamy virus] | 162 | 162 | 48% | 1.00E-38 | 28% | CAB54100.1 |
| env protein, surface domain [Simian foamy virus] | 162 | 162 | 53% | 1.00E-38 | 28% | CAB54108.1 |
| env protein, surface domain [Simian foamy virus] | 161 | 161 | 48% | 3.00E-38 | 28% | CAB54097.1 |
| env protein, surface domain [Simian foamy virus] >emb\|CAB54094.1\| env protein, surface domain [Simian foamy virus] | 160 | 160 | 48% | 3.00E-38 | 29% | CAB54091.1 |
| env protein, surface domain [Simian foamy virus] >emb\|CAB54112.1\| env protein, surface domain [Simian foamy virus] | 160 | 160 | 48% | 4.00E-38 | 28% | CAB54111.1 |
| env protein, surface domain [Simian foamy virus] | 160 | 160 | 48% | 6.00E-38 | 28% | CAB54092.1 |
| env protein, surface domain [Simian foamy virus] | 159 | 159 | 48% | 1.00E-37 | 28% | CAB54123.1 |
| env protein, surface domain [Simian foamy virus] | 159 | 159 | 48% | 2.00E-37 | 28% | CAB54116.1 |
| env protein, surface domain [Simian foamy virus] | 159 | 159 | 48% | 2.00E-37 | 28% | CAB54095.1 |
| env protein, surface domain [Simian foamy virus] | 158 | 158 | 48% | 2.00E-37 | 28% | CAB54113.1 |
| env protein, surface domain [Simian foamy virus] | 153 | 153 | 48% | 1.00E-35 | 28% | CAB54103.1 |
| env protein, surface domain [Simian foamy virus] | 153 | 153 | 48% | 1.00E-35 | 28% | CAB54105.1 |
| env protein, surface domain [Simian foamy virus] | 143 | 143 | 53% | 2.00E-32 | 27% | CAB54109.1 |
| envelope protein [Human spumaretrovirus] | 128 | 128 | 23% | 2.00E-29 | 34% | AAF78576.1 |
| envelope protein [Human spumaretrovirus] | 125 | 125 | 23% | 1.00E-28 | 34% | AAF78578.1 |
| envelope [Simian foamy virus] | 82.8 | 82.8 | 12% | 1.00E-14 | 33% | ABA18730.2 |
| envelope [Simian foamy virus] | 82 | 82 | 12% | 2.00E-14 | 34% | ABA18727.2 |
| envelope [Simian foamy virus] | 80.9 | 80.9 | 11% | 5.00E-14 | 34% | ABA18729.1 |
| envelope [Simian foamy virus] | 80.9 | 80.9 | 12% | 5.00E-14 | 34% | ABA18731.2 |
| envelope [Simian foamy virus] | 80.5 | 80.5 | 11% | 7.00E-14 | 34% | ABA18728.1 |
| envelope [Simian foamy virus] | 79.7 | 79.7 | 11% | 1.00E-13 | 34% | ABA18735.1 |
| envelope [Simian foamy virus] | 79.7 | 79.7 | 11% | 1.00E-13 | 34% | ABA18732.1 |
| envelope [Simian foamy virus] | 79.3 | 79.3 | 11% | 1.00E-13 | 34% | ABA18733.1 |
| envelope [Simian foamy virus] | 79 | 79 | 11% | 2.00E-13 | 34% | ABA18736.1 |
| envelope [Simian foamy virus] | 79 | 79 | 11% | 2.00E-13 | 34% | ABA18734.1 |
| envelope protein, partial [Bovine foamy virus] >gb\|AFQ41114.1\| envelope protein, partial [Bovine foamy virus] >gb\|AFQ41115.1\| envelope protein, partial [Bovine foamy virus] | 77.4 | 77.4 | 24% | 3.00E-12 | 28% | AFQ41113.1 |
| envelope protein, partial [Bovine foamy virus] | 76.6 | 76.6 | 23% | 7.00E-12 | 28% | AFQ41116.1 |
| envelope protein, partial [Macaque simian foamy virus] | 62.8 | 62.8 | 8% | 3.00E-08 | 41% | AAA47801.1 |
