# Supplementary material for: Endogenous Viral Sequences from the Cape Golden Mole (Chrysochloris asiatica) Reveal the Presence of Foamy Viruses in All Major Placental Mammal Clades
Source: PLoS One. 2014 May 16;9(5):e97931. doi: 10.1371/journal.pone.0097931 (PMC4024004; doi:10.1371/journal.pone.0097931)
Supplement: Table S2 — PSI-BLAST results using CaEFV Env protein as a query (E value threshold of 0.01). (DOCX) [file pone.0097931.s002.docx]

**Table S2 PSI-BLAST results using CaEFV Env protein as a query (E value threshold of 0.01)**

| **Description [taxon]** | **Max score** | **Total score** | **Query cover** | **E value** | **Identity** | **Accession** |
| --- | --- | --- | --- | --- | --- | --- |
| env protein [Macaque simian foamy virus] | 1435 | 1435 | 99% | 0 | 27% | AGM61339.1 |
| Env [Macaque simian foamy virus] >sp\|P23073.3\|ENV_SFV1 RecName: Full=Envelope glycoprotein gp130; AltName: Full=Env polyprotein; Contains: RecName: Full=Leader peptide; Short=LP; AltName: Full=Env leader protein; Short=Elp; AltName: Full=gp18LP; Contains: RecName: Full=Surface protein; Short=SU; AltName: Full=Glycoprotein 80; Short=gp80; Contains: RecName: Full=Transmembrane protein; Short=TM; AltName: Full=Glycoprotein 48; Short=gp48 | 1420 | 1420 | 99% | 0 | 27% | YP_001961123.1 |
| envelope protein [Simian foamy virus] | 1417 | 1417 | 99% | 0 | 27% | AAA47794.1 |
| envelope protein [Macaque simian foamy virus] | 1411 | 1411 | 99% | 0 | 28% | AFA44810.1 |
| env [Human foamy virus] >emb\|CAA68994.1\| env [Human foamy virus] >emb\|CAA69000.1\| env [Human foamy virus] | 1411 | 1411 | 99% | 0 | 28% | CAA69004.1 |
| RecName: Full=Envelope glycoprotein gp130; AltName: Full=Env polyprotein; Contains: RecName: Full=Leader peptide; Short=LP; AltName: Full=Env leader protein; Short=Elp; AltName: Full=gp18LP; Contains: RecName: Full=Surface protein; Short=SU; AltName: Full=Glycoprotein 80; Short=gp80; Contains: RecName: Full=Transmembrane protein; Short=TM; AltName: Full=Glycoprotein 48; Short=gp48 >gb\|AAB48113.1\| envelope protein [Human spumaretrovirus] | 1406 | 1406 | 99% | 0 | 28% | P14351.2 |
| env polyprotein - human foamy virus >emb\|CAA29086.1\| unnamed protein product [Human spumaretrovirus] >gb\|AAA46123.1\| envelope protein [Human spumaretrovirus] | 1406 | 1406 | 99% | 0 | 28% | VCLJSP |
| envelope protein [Feline foamy virus] | 1402 | 1402 | 99% | 0 | 28% | BAB33175.1 |
| env protein [Macaque simian foamy virus] | 1394 | 1394 | 99% | 0 | 27% | AGM61337.1 |
| envelope protein, partial [Simian foamy virus 3] | 1393 | 1393 | 99% | 0 | 28% | AAA47798.1 |
| Env [African green monkey simian foamy virus] >sp\|P27399.1\|ENV_SFV3L RecName: Full=Envelope glycoprotein gp130; AltName: Full=Env polyprotein; Contains: RecName: Full=Leader peptide; Short=LP; AltName: Full=Env leader protein; Short=Elp; AltName: Full=gp18LP; Contains: RecName: Full=Surface protein; Short=SU; AltName: Full=Glycoprotein 80; Short=gp80; Contains: RecName: Full=Transmembrane protein; Short=TM; AltName: Full=Glycoprotein 48; Short=gp48 >pir\|\|VCLJLK env polyprotein - simian foamy virus (type 3, strain LK3) | 1393 | 1393 | 99% | 0 | 28% | YP_001956723.2 |
| env protein [Macaque simian foamy virus] | 1393 | 1393 | 99% | 0 | 27% | AGM61343.1 |
| env protein [Simian foamy virus] | 1391 | 1391 | 99% | 0 | 29% | AFX98100.1 |
| env protein [Simian foamy virus] | 1387 | 1387 | 99% | 0 | 27% | AFX98085.1 |
| envelope protein [Simian foamy virus] >sp\|Q87041.1\|ENV_SFVCP RecName: Full=Envelope glycoprotein gp130; AltName: Full=Env polyprotein; Contains: RecName: Full=Leader peptide; Short=LP; AltName: Full=Env leader protein; Short=Elp; AltName: Full=gp18LP; Contains: RecName: Full=Surface protein; Short=SU; AltName: Full=Glycoprotein 80; Short=gp80; Contains: RecName: Full=Transmembrane protein; Short=TM; AltName: Full=Glycoprotein 48; Short=gp48 >gb\|AAA19979.1\| env [Simian foamy virus] | 1383 | 1383 | 99% | 0 | 27% | NP_056804.1 |
| env protein [Simian foamy virus] | 1382 | 1382 | 99% | 0 | 28% | AFX98080.1 |
| env protein [Simian foamy virus] | 1363 | 1363 | 99% | 0 | 27% | AFX98095.1 |
| envelope protein [Simian foamy virus-gorilla] | 1362 | 1362 | 99% | 0 | 27% | ADN65592.1 |
| env protein [Simian foamy virus] | 1361 | 1361 | 99% | 0 | 27% | AFX98090.1 |
| envelope glycoprotein [Simian foamy virus-orangutan] | 1331 | 1331 | 99% | 0 | 27% | CAD67563.1 |
| env [Spider monkey foamy virus] | 1215 | 1215 | 99% | 0 | 27% | ABV59400.1 |
| env [Squirrel monkey foamy virus] | 1207 | 1207 | 99% | 0 | 28% | ADE05996.1 |
| env [Common marmoset foamy virus] | 1204 | 1204 | 99% | 0 | 27% | ADE06001.1 |
| envelope protein [Bovine foamy virus] | 1196 | 1196 | 99% | 0 | 28% | AFR79245.1 |
| envelope protein [Bovine foamy virus] | 1195 | 1195 | 96% | 0 | 28% | AFR79240.1 |
| envelope protein [Bovine foamy virus] | 1182 | 1182 | 96% | 0 | 28% | AAN08117.1 |
| envelope protein [Bovine foamy virus] >gb\|AAB68771.1\| envelope protein [Bovine foamy virus] | 1181 | 1181 | 99% | 0 | 28% | NP_044930.1 |
| Env [Feline foamy virus] | 1158 | 1158 | 99% | 0 | 26% | AGC11914.1 |
| Env [Feline foamy virus] | 1156 | 1156 | 99% | 0 | 26% | AGC11909.1 |
| Env [equine foamy virus] >gb\|AAF64415.1\|AF201902_3 Env [equine foamy virus] | 1148 | 1148 | 99% | 0 | 28% | NP_054717.1 |
| envelope protein [Feline foamy virus] | 1126 | 1126 | 99% | 0 | 27% | BAB33173.1 |
| hypothetical protein FFV_gp1 [Feline foamy virus] >sp\|O56861.1\|ENV_FFV RecName: Full=Envelope glycoprotein gp130; AltName: Full=Env polyprotein; Contains: RecName: Full=Leader peptide; Short=LP; AltName: Full=Env leader protein; Short=Elp; AltName: Full=gp18LP; Contains: RecName: Full=Surface protein; Short=SU; AltName: Full=Glycoprotein 80; Short=gp80; Contains: RecName: Full=Transmembrane protein; Short=TM; AltName: Full=Glycoprotein 48; Short=gp48 >emb\|CAA70076.1\| env [Feline foamy virus] >emb\|CAA11582.1\| env [Feline foamy virus] | 1126 | 1126 | 99% | 0 | 26% | NP_056915.1 |
| FeSFV envelope [Feline foamy virus] >gb\|AAC58532.1\| envelope protein [Feline foamy virus] >emb\|CAD92797.1\| envelope protein [Feline foamy virus] | 1120 | 1120 | 99% | 0 | 26% | AAB38322.1 |
| envelope protein [Feline foamy virus] | 1120 | 1120 | 99% | 0 | 26% | CAD92801.1 |
| envelope protein [Feline foamy virus] | 1116 | 1116 | 99% | 0 | 27% | BAB33174.1 |
| env protein, surface domain [Simian foamy virus] | 826 | 826 | 59% | 0 | 26% | CAB54108.1 |
| env protein, surface domain [Simian foamy virus] | 825 | 825 | 59% | 0 | 26% | CAB54106.1 |
| env protein, surface domain [Simian foamy virus] | 819 | 819 | 59% | 0 | 26% | CAB54088.1 |
| env protein, surface domain [Simian foamy virus] | 815 | 815 | 59% | 0 | 25% | CAB54083.1 |
| env protein, surface domain [Simian foamy virus] | 814 | 814 | 59% | 0 | 25% | CAB54084.1 |
| env protein, surface domain [Simian foamy virus] | 813 | 813 | 59% | 0 | 26% | CAB54122.1 |
| env protein, surface domain [Simian foamy virus] | 813 | 813 | 59% | 0 | 25% | CAB54086.1 |
| env protein, surface domain [Simian foamy virus] >emb\|CAB54090.1\| env protein, surface domain [Simian foamy virus] | 813 | 813 | 59% | 0 | 25% | CAB54089.1 |
| env protein, surface domain [Simian foamy virus] | 813 | 813 | 59% | 0 | 25% | CAB54082.1 |
| env protein, surface domain [Simian foamy virus] | 813 | 813 | 59% | 0 | 26% | CAB54128.1 |
| env protein, surface domain [Simian foamy virus] >emb\|CAB54117.1\| env protein, surface domain [Simian foamy virus] >emb\|CAB54120.1\| env protein, surface domain [Simian foamy virus] >emb\|CAB54125.1\| env protein, surface domain [Simian foamy virus] >emb\|CAB54129.1\| env protein, surface domain [Simian foamy virus] | 813 | 813 | 59% | 0 | 26% | CAB54099.1 |
| env protein, surface domain [Simian foamy virus] | 812 | 812 | 59% | 0 | 26% | CAB54118.1 |
| env protein, surface domain [Simian foamy virus] | 812 | 812 | 59% | 0 | 26% | CAB54115.1 |
| env protein, surface domain [Simian foamy virus] | 812 | 812 | 59% | 0 | 26% | CAB54102.1 |
| env protein, surface domain [Simian foamy virus] | 811 | 811 | 59% | 0 | 25% | CAB54119.1 |
| env protein, surface domain [Simian foamy virus] | 811 | 811 | 59% | 0 | 26% | CAB54096.1 |
| env protein, surface domain [Simian foamy virus] | 811 | 811 | 59% | 0 | 26% | CAB54100.1 |
| env protein, surface domain [Simian foamy virus] | 811 | 811 | 59% | 0 | 25% | CAB54093.1 |
| env protein, surface domain [Simian foamy virus] | 811 | 811 | 59% | 0 | 25% | CAB54127.1 |
| env protein, surface domain [Simian foamy virus] | 811 | 811 | 59% | 0 | 26% | CAB54101.1 |
| env protein, surface domain [Simian foamy virus] >emb\|CAB54094.1\| env protein, surface domain [Simian foamy virus] | 811 | 811 | 59% | 0 | 25% | CAB54091.1 |
| env protein, surface domain [Simian foamy virus] | 811 | 811 | 59% | 0 | 26% | CAB54126.1 |
| env protein, surface domain [Simian foamy virus] | 810 | 810 | 59% | 0 | 26% | CAB54124.1 |
| env protein, surface domain [Simian foamy virus] | 810 | 810 | 59% | 0 | 26% | CAB54085.1 |
| env protein, surface domain [Simian foamy virus] | 810 | 810 | 59% | 0 | 26% | CAB54121.1 |
| env protein, surface domain [Simian foamy virus] | 810 | 810 | 59% | 0 | 25% | CAB54097.1 |
| env protein, surface domain [Simian foamy virus] >emb\|CAB54112.1\| env protein, surface domain [Simian foamy virus] | 810 | 810 | 59% | 0 | 25% | CAB54111.1 |
| env protein, surface domain [Simian foamy virus] | 809 | 809 | 59% | 0 | 25% | CAB54092.1 |
| env protein, surface domain [Simian foamy virus] | 809 | 809 | 59% | 0 | 26% | CAB54130.1 |
| env protein, surface domain [Simian foamy virus] | 808 | 808 | 59% | 0 | 26% | CAB54114.1 |
| env protein, surface domain [Simian foamy virus] | 808 | 808 | 59% | 0 | 26% | CAB54095.1 |
| env protein, surface domain [Simian foamy virus] | 808 | 808 | 59% | 0 | 25% | CAB54110.1 |
| env protein, surface domain [Simian foamy virus] | 808 | 808 | 59% | 0 | 25% | CAB54123.1 |
| env protein, surface domain [Simian foamy virus] | 807 | 807 | 59% | 0 | 25% | CAB54113.1 |
| env protein, surface domain [Simian foamy virus] | 807 | 807 | 59% | 0 | 25% | CAB54087.1 |
| env protein, surface domain [Simian foamy virus] | 807 | 807 | 59% | 0 | 26% | CAB54104.1 |
| env protein, surface domain [Simian foamy virus] | 804 | 804 | 59% | 0 | 25% | CAB54116.1 |
| env protein, surface domain [Simian foamy virus] | 799 | 799 | 59% | 0 | 26% | CAB54105.1 |
| env protein, surface domain [Simian foamy virus] | 793 | 793 | 59% | 0 | 26% | CAB54103.1 |
| env protein, surface domain [Simian foamy virus] | 790 | 790 | 59% | 0 | 26% | CAB54109.1 |
| envelope protein, partial [Bovine foamy virus] | 687 | 687 | 59% | 0 | 26% | ABM55470.1 |
| envelope protein, partial [Bovine foamy virus] | 686 | 686 | 59% | 0 | 26% | ABM55473.1 |
| envelope protein, partial [Bovine foamy virus] | 684 | 684 | 59% | 0 | 26% | ABM55472.1 |
| envelope protein, partial [Bovine foamy virus] | 682 | 682 | 59% | 0 | 26% | ABM55471.1 |
| envelope protein, partial [Bovine foamy virus] | 676 | 676 | 59% | 0 | 26% | ABM55474.1 |
| envelope protein, partial [Human spumaretrovirus] | 521 | 521 | 35% | 6.00E-174 | 30% | AAF00493.1 |
| envelope glycoprotein, partial [Rhinolophus affinis foamy virus 1] | 516 | 516 | 48% | 4.00E-170 | 26% | AFK85016.1 |
| envelope protein [Human spumaretrovirus] | 339 | 339 | 23% | 1.00E-105 | 32% | AAF78576.1 |
| envelope protein [Human spumaretrovirus] | 336 | 336 | 23% | 2.00E-104 | 32% | AAF78578.1 |
| envelope protein, partial [Bovine foamy virus] >gb\|AFQ41114.1\| envelope protein, partial [Bovine foamy virus] >gb\|AFQ41115.1\| envelope protein, partial [Bovine foamy virus] | 329 | 329 | 28% | 3.00E-101 | 23% | AFQ41113.1 |
| envelope protein, partial [Bovine foamy virus] | 326 | 326 | 28% | 6.00E-100 | 23% | AFQ41116.1 |
| envelope [Simian foamy virus] | 228 | 228 | 14% | 3.00E-66 | 31% | ABA18731.2 |
| envelope [Simian foamy virus] | 227 | 227 | 14% | 4.00E-66 | 31% | ABA18727.2 |
| envelope [Simian foamy virus] | 225 | 225 | 14% | 2.00E-65 | 30% | ABA18730.2 |
| envelope [Simian foamy virus] | 221 | 221 | 13% | 9.00E-64 | 31% | ABA18733.1 |
| envelope [Simian foamy virus] | 220 | 220 | 13% | 2.00E-63 | 31% | ABA18732.1 |
| envelope [Simian foamy virus] | 219 | 219 | 13% | 3.00E-63 | 32% | ABA18729.1 |
| envelope [Simian foamy virus] | 218 | 218 | 13% | 7.00E-63 | 31% | ABA18728.1 |
| envelope [Simian foamy virus] | 218 | 218 | 13% | 1.00E-62 | 30% | ABA18735.1 |
| envelope [Simian foamy virus] | 217 | 217 | 13% | 2.00E-62 | 32% | ABA18734.1 |
| envelope [Simian foamy virus] | 203 | 203 | 12% | 2.00E-57 | 31% | ABA18736.1 |
| envelope protein [Feline foamy virus] | 195 | 195 | 20% | 1.00E-53 | 20% | AAC17996.1 |
| envelope protein, partial [Macaque simian foamy virus] | 150 | 150 | 9% | 5.00E-39 | 34% | AAA47801.1 |
